# Torsion of the greater omentum: two case reports

**DOI:** 10.1186/s13256-015-0641-5

**Published:** 2015-07-11

**Authors:** Konstantinos Alexiou, Argyrios Ioannidis, Ioannis Drikos, Nicolaos Sikalias, Nicolaos Economou

**Affiliations:** Department of Surgery, Sismanoglion General Hospital, Sismanoglou 1, P.O. BOX 15126, Athens, Greece

**Keywords:** Great omentum, Laparoscopy, Omental torsion

## Abstract

**Introduction:**

Torsion of the omentum is a benign self-limiting disorder, which is difficult to diagnose because the main symptoms are similar to those of other abdominal diseases. Most of the published cases had been diagnosed during operation via direct eye view. According to several studies, it is important that the correct preoperative diagnosis is made as omental torsion can be treated conservatively in most cases without any complications avoiding surgical intervention. However, patients should be under clinical and laboratory observation in order to detect symptoms that would lead to surgical intervention in which case a laparoscopy is the appropriate surgical treatment.

**Case presentation:**

Torsion of the great omentum is a rare cause of acute abdominal pain which is usually misdiagnosed. In this study we report two cases, a 52-year-old Greek woman and a 68-year-old Greek man, who presented at our emergency room with symptoms such as right lower quadrant pain and tenderness similar to acute appendicitis. In both cases a surgical exploratory laparotomy of the abdomen revealed a twisted heavily congested segment of the right part of the greater omentum accompanied by intra-abdominal serosanguinous fluid.

**Conclusions:**

Greater omental torsion is difficult to diagnose preoperatively. It presents as acute abdominal pain located more often in the right iliac fossa. It is very important to make a correct preoperative diagnosis because omental torsion is a benign self-limiting disorder that can be treated conservatively, avoiding laparotomy. When a patient’s clinical, laboratory and radiological findings worsen or diagnosis is doubtful then laparoscopy is the appropriate method for diagnosis and treatment.

## Introduction

Omental torsion is a rare cause of acute abdominal pain [1]; diagnosis of omental torsion is usually difficult because clinical signs and symptoms are similar to other common causes of abdominal pain. The most common preoperative diagnosis is acute appendicitis and the proper preoperative diagnosis is important for the appropriate treatment option [2]. Diagnosis of omental torsion is difficult and mainly based on ultrasound and computed tomography (CT) scan analysis [3].

Omental torsion was first described by Eitel in 1899. Bason and Jones analyzed 223 cases of primary torsion and revealed that only one patient had been correctly diagnosed preoperatively [4]. Since then, 54 more cases have been published and still only a few of them had been diagnosed correctly.

The primary form of omentum torsion is usually caused by rotation of the movable portion of the omentum and is influenced by several factors [5]. Causes of torsion of the great omentum include anatomical variations, such as bifurcated omentum, obesity and alterations in blood circulation [6, 7]. The main precipitating factors affecting omentum torsion include cough, sudden change in body position, especially in cases of increased bowel motion in a compressed state between the liver and the abdominal wall [6, 7]. Secondary torsion is more common and occurs in most cases of omental torsion. This form is often associated with adhesions of the omentum, which can be caused by hernias, postoperative wounds, tumors and intraventricular inflammations [6, 7]. Torsion of the greater omentum often appears on the right side mainly due to the larger size and mobility of the omentum. The torsion may include part of the omentum or the entire length [6, 7].

Torsion of the great omentum is difficult to diagnose preoperatively with pinpoint accuracy limited to rates of 0.6 % to 4.8 % of all cases. Clinical manifestations include sudden pain especially in the side where the torsion is located and the stimulation of the abdominal wall seems to cause peritoneal irritation [7, 8]. Other nonspecific symptoms may appear such as nausea, vomiting, fever and leukocytosis [7, 8].

The major complications of omental torsion include rupture and intraperitoneal bleeding, filtration purulent peritonitis and intraperitoneal abscess while fibrosis and inflammatory reaction may occur at occlusion. The diagnosis is difficult and requires confirmation process to display. Abdominal ultrasound as a diagnostic method may exclude from the differential diagnosis conditions such as cholecystitis [9]. The diagnosis is usually made by laparoscopy especially in cases where there is evidence of suspected appendicitis, which can be referred to 83 % of cases. During the operation, more common causes of abdominal pain such as appendicitis, diverticulum perforation, Meckel’s diverticulum and ovarian cancer, are excluded [9, 10].

In this case report we analyze two cases of greater omental torsion in a 52-year-old Greek woman and a 68-year-old Greek man who presented at our emergency room (ER) with clinical symptoms and laboratory findings of acute appendicitis. They underwent laparotomy where the diagnosis of greater omental torsion was made. The appendix in both cases was normal and they had no other disorders. Due to the rarity and the difficulty of diagnosis we present the current aspects of diagnosis and treatment of greater omental torsion.

## Case presentation

Case 1 was a 52-year-old Greek woman who presented at our ER referring abdominal pain at the right iliac fossa. There was no history of any previous abdominal operations or signs of recent infections.

A physical examination revealed slightly rigid pain at the lower part of her abdomen and especially at the right iliac fossa with positive McBurney point, rebound tenderness and Rovsing sign. Her abdominal sounds were diminished. A clinical examination revealed normal cervix and appendages without any palpable space; her blood pressure, pulses and body temperature were normal.

Her laboratory findings were white blood cells 8.81μL (normal 4000 to 10000), neutrophils 47.7 % (45 to 75 %), lymphocytes 35.7 % (normal 16 to 46 %), red blood cells (RBC) 4.20M/mL (normal 4.0 to 5.2), hemoglobin (Hb) 11.6g/dL (12.3 to 15.7g/dL), hematocrit (Hct) 36.3 % (normal 37 to 46 %), platelets (PLT) 288K/uL (normal 150 to 400), blood glucose 100mg/dL (normal 70 to 112), blood urea nitrogen 49mg/dL (normal 10 to 50), creatinine 0.7mg/dL (normal 0.5 to 1.2), total bilirubin 0.4mg/dL (normal 0.3 to 1.5), direct bilirubin 0.1mg/dL (normal 0.01 to 0.35), aspartate aminotransferase 18U/L (normal 0 to 40), alanine aminotransferase 10U/L (normal 0 to 40), lactate dehydrogenase 126U/L (normal 105 to 333), creatine kinase 55U/L (55 to 160), sodium 140mmol/L (normal 135 to 153), potassium 4.3mmol/L (normal 3.5 to 5.3), and amylase 73U/L (normal 40 to 140). Urine tests revealed PH acidic, Hb traces; WBC 1 to 3, RBC 1 to 3 and urine amylase 216U/L. The chest and abdominal X-rays were normal.

**Fig. 1:**
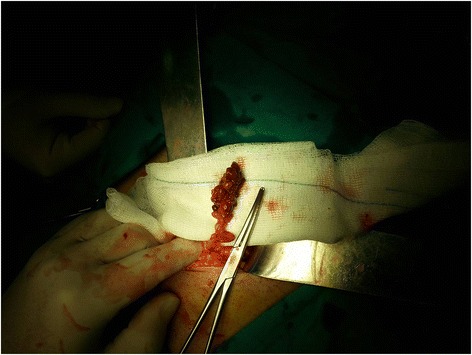
The affected portion of the omentum, twisted many times in a clockwise direction free of any adhesions

Due to intense clinical signs and worsening of the symptoms the patient underwent an operation with the probable diagnosis of acute appendicitis. In this case a laparotomy with a McBurney incision was performed. A small amount of intra-abdominal serosanguinous fluid was present and revealed a dark red congested area, with vessel thrombosis and necrotic omental mass. Her appendix was normal. The affected portion of her omentum was on the right distal free edge, twisted many times in a clockwise direction free of any adhesions (Fig. 1).

The operation proceeded with resection of the affected part of her omentum. Exploration of any other possible abdominal diseases was also performed even if clinical signs and symptoms were negative. An appendectomy was performed. She recovered well with immediate resolution of the symptoms and without any postoperative complications.

Case 2 was a 68-year-old Greek man who presented at our ER with abdominal pain and high sensitivity at the right iliac fossa. There was no history of any previous abdominal operations.

A physical examination revealed rigid abdomen with positive McBurney point, rebound tenderness and Rovsing sign. His bowel sounds were diminished to absent. His blood pressure was 140/85mmHg, pulse 80 beats/minute and body temperature 37.5 °C. His laboratory findings were WBC 14.000 (normal 4000 to 10000), neutrophils 79.4 % (45 to 75 %), lymphocytes 16.6 % (normal 16 to 46 %), RBC 5.20M/mL (normal 5.0 to 6.2), Hb 12.3g/dL (13.3 to 16.4g/dL), Hct 36.3 % (40 to 52 %), and PLT 320K/uL (150 to 400). The results of the rest of the biochemical tests, and chest and abdominal X-rays were normal.

Diagnosis of acute appendicitis was so profound that abdominal ultrasound and CT scan were not performed. The patient underwent laparotomy with a McBurney incision. There was a large amount of bloody stained serous fluid present in his abdomen. A large segment of his greater omentum (18 × 10cm) was found twisted around its base and was loosely attached to his underlying right colon. The excised segment had a brisk red to gray color and it was heavily congested with multiple hemorrhagic infiltrations; these findings were also stated in a histological examination.

The surgical operation proceeded with omentectomy of the affected segment. The exploration of the rest of his abdominal cavity showed no other abnormalities and revealed a normal appendix. He recovered well, with a postoperative period free of complications and symptoms.

## Discussion

Torsion of the greater omentum is a rare clinical condition which is caused by twisting of the omentum around a pivotal point, more often in a clockwise direction; it is associated with ischemia and acute abdominal pain. Omental infarction is a benign self-limiting disorder [2, 10, 11] which in unremitting cases could cause ischemic necrosis of the involved part of the omentum and in intermittent cases may be a rare cause of recurrent abdominal pain [11].

Omental torsion may be primary, in which there is no obvious cause, or secondary, where it can be caused by several factors [12]. Secondary torsion is more common than primary torsion. Secondary torsion is divided into unipolar, where the free end of the omentum is twisted, and bipolar, where the omentum is twisted between its base and another fixed area, such as adhesions to the free end [12]. Unipolar omental torsion is associated with conditions such as cysts and tumors [13] of the omentum [14] or an external and internal hernia [15].

Primary forms occur more often in the third and fifth decade of life and are more common in men than women [16]. Omental torsion may also occur in children although the greater omentum is incompletely developed in childhood. The left segment of the omentum is less frequently involved at the torsion than the right because the right segment is more mobile due to its greater length and weight. As the omentum twists, the compromised venous system causes aseptic peritonitis with serosanguinous fluid accumulation in the peritoneal cavity. Furthermore, the arteries are compromised causing necrosis of the affected part. If torsion of the greater omentum is left untreated, it may form a fibrous mass surrounded by adhesions which could become infected or it may be separated inside the abdominal cavity.

Omental torsion has no certain etiology and sometimes it is idiopathic. Possible causes of primary torsion include long omentum, long pedicle of omentum, and relative increase in the bulk of the distal portion of the omentum by fatty deposition or bifid omentum [17]. Mechanisms initiating torsion include trauma, obesity, physical activity, coughing, pregnancy [17, 18], abdominal surgery [17, 18] and constant movement of the omentum by peristalsis. In both cases presented in this study, there was no identifiable cause of omental torsion while the omentum of Patient 2 was in bifid condition and it could be associated with omental torsion.

Omental torsion is difficult to diagnose preoperatively [12]. It presents as acute unremitting abdominal pain located more often in the right iliac fossa similar to acute appendicitis or to the right abdominal side mimicking acute cholecystitis, pancreatitis and perforated duodenal ulcer [12].

Less often, pain is located on the right hypochondrium revealed as acute cholecystitis [3, 19]. When the left part of the omentum is involved, the clinical signs resemble diverticulitis [11, 19]. This entity is very rare for the left segment of the omentum because this part is less frequently involved due to less movement.

On examination guarding rebound tenderness is frequently presented in the right iliac fossa but away from the McBurney’s point [20, 21]. A mobile tender mass is noted in one-third of cases. Nausea and vomiting are sometimes present even if the periumbilical pain of acute appendicitis is absent [21–23].

Leukocytosis with increased levels of C-reactive protein of the plasma and elevated erythrocyte sedimentation rate are nearly always present [2, 24], while fever is a less constant sign. An abdominal X-ray is nonspecific in most cases of omental torsion [25]. Ultrasound imaging shows a hyperechoic, noncompressible, ovoid mass that is adherent to the abdominal wall [26]. On CT scan a well-circumscribed mass is revealed composed of fat interspersed with hyperattenuating streaks [26]. The presence of the vascular pedicle is a reliable diagnostic sign of omental torsion [14] which may specify torsion from other tumors of the omentum [25, 26]. Ultrasound and CT scan analysis are very sensitive for the preoperative diagnosis of torsion of the greater omentum in the absence of other abdominal signs [8].

According to the literature it is very important to make a correct preoperative diagnosis because omental torsion is a benign self-limiting disorder and it is proposed that it can be treated conservatively, avoiding laparotomy [8]. Van Breda Vriesman *et al*. reported on the cases of 40 patients with epiploic appendicitis and omental torsion. All patients recovered well under conservative treatment without any complications [27]. Patients under conservative treatment should be under continuous clinical and radiological observation [27]. When the patient’s clinical, laboratory and radiological findings worsen or when diagnosis is doubtful or the surgeon decides that surgical intervention is required, then laparoscopy is the appropriate method for diagnosis and therapy [28, 29].

CT scan analysis was not performed in our cases because the clinical signs were very typical for acute appendicitis, especially in Patient 2 where the clinical signs were accompanied by low grade fever and mild leukocytosis. An ultrasound scan was performed for Patient 1 and free abdominal fluid was recognized. Even if omental infarction is not usually diagnosed preoperatively almost all the published cases had been diagnosed after laparotomy.

Surgical exploration reveals serosanguinous fluid, a normal appendix and the hemorrhagic necrotic portion of the greater omentum. The affected omental portion should be excised; the clinical signs and symptoms subside immediately after omentectomy.

## Conclusions

Greater omental torsion is difficult to diagnose preoperatively. It presents as acute abdominal pain located more often in the right iliac fossa. Omental torsion is a benign self-limiting disorder and in most cases can be treated conservatively avoiding laparotomy. When the patient’s clinical, laboratory and radiological findings worsen or when diagnosis is doubtful surgical intervention such as laparoscopy is the proper method for diagnosis and treatment.

## Consent

Written informed consent was obtained from the patients for publication of this case report and any accompanying images. A copy of the written consents is available for review by the Editor-in-Chief of this journal.

## Abbreviations

ER: Emergency room
CT: Computed tomography
Hb: Hemoglobin
Hct: Hematocrit
PLT: Platelets
RBC: Red blood cells
WBC: White blood cells
